# Quantification of Errors in Ordinal Outcome Scales Using Shannon Entropy: Effect on Sample Size Calculations

**DOI:** 10.1371/journal.pone.0067754

**Published:** 2013-07-05

**Authors:** Pitchaiah Mandava, Chase S. Krumpelman, Jharna N. Shah, Donna L. White, Thomas A. Kent

**Affiliations:** 1 The Michael E. DeBakey Veterans Affairs Medical Center Comprehensive Stroke Program, Houston, Texas, United States of America; 2 Clinical Epidemiology and Comparative Effectiveness Program, Houston Veterans Affairs Health Services Research Center of Excellence, Houston, Texas, United States of America; 3 The Stroke Outcomes Laboratory, Department of Neurology, Baylor College of Medicine, Houston, Texas, United States of America; Cardiff University, United Kingdom

## Abstract

**Objective:**

Clinical trial outcomes often involve an ordinal scale of subjective functional assessments but the optimal way to quantify results is not clear. In stroke, the most commonly used scale, the modified Rankin Score (mRS), a range of scores (“Shift”) is proposed as superior to dichotomization because of greater information transfer. The influence of known uncertainties in mRS assessment has not been quantified. We hypothesized that errors caused by uncertainties could be quantified by applying information theory. Using Shannon’s model, we quantified errors of the “Shift” compared to dichotomized outcomes using published distributions of mRS uncertainties and applied this model to clinical trials.

**Methods:**

We identified 35 randomized stroke trials that met inclusion criteria. Each trial’s mRS distribution was multiplied with the noise distribution from published mRS inter-rater variability to generate an error percentage for “shift” and dichotomized cut-points. For the SAINT I neuroprotectant trial, considered positive by “shift” mRS while the larger follow-up SAINT II trial was negative, we recalculated sample size required if classification uncertainty was taken into account.

**Results:**

Considering the full mRS range, error rate was 26.1%±5.31 (Mean±SD). Error rates were lower for all dichotomizations tested using cut-points (e.g. mRS 1; 6.8%±2.89; overall p<0.001). Taking errors into account, SAINT I would have required 24% more subjects than were randomized.

**Conclusion:**

We show when uncertainty in assessments is considered, the lowest error rates are with dichotomization. While using the full range of mRS is conceptually appealing, a gain of information is counter-balanced by a decrease in reliability. The resultant errors need to be considered since sample size may otherwise be underestimated. In principle, we have outlined an approach to error estimation for any condition in which there are uncertainties in outcome assessment. We provide the user with programs to calculate and incorporate errors into sample size estimation.

## Introduction

In the analysis of new therapeutic approaches to disease, it is essential that the effects of treatment be captured in a reliable manner. Measures for many conditions include scales that involve subjective assessment of a subject’s well-being comparing two different treatments. In the case of stroke, the modified Rankin Score (mRS) is the most widely adopted measure of recovery of function in stroke trials [1]. As an ordinal scale, this instrument provides an ordering of possible outcomes, ranging from near complete recovery (e.g., 0 in mRS) to death (e.g., 6 in mRS). Analysis of outcome results can be performed by two methods: 1) Full-scale analysis where results for each group (treatment and placebo) are depicted as a proportion of patients in some or all ascending grade, and, 2) “Dichotomization” where results for each group are depicted as proportion of patients into two collapsed or binned grade categories (e.g. mRS 0–1 indicating excellent recovery, mRS 2–5, a dependant state), with an added “safety” category of mortality (mRS 6).

Dichotomization of outcome scales including dichotomization of mRS at cut-point of 1 (e.g, mRS 0–1 vs. 2–6) was used successfully in the NINDS trial of intravenous alteplase for ischemic stroke [2]. Of note alteplase is the first and only medication approved by FDA for use in ischemic stroke. More recently dichotomization at higher cut-points of mRS 3 and 4 have been employed in three randomized stroke trials of hemicraniectomy (DECIMAL, DESTINY, and HAMLET), which had patients with high baseline stroke severity, all of which were positive with relatively low number of subjects [3]–[5].

There remains discussion as to which method of analysis is the most appropriate approach for outcome measures in stroke trials. For example, the European Medical Agency issued guidance that when ordinal scales are used for testing the efficacy of novel medicines or devices, the full-scale be analyzed [6]. The impetus for this guidance came from the work of Whitehead [7], and Campbell et al [8], which showed that when number of categories is increased from two to six, sample size requirements are reduced by 23% because of a gain in the amount of information available [9]. Along these lines, several authors have suggested abandoning dichotomization in favor of ordinal scale analysis [10]–[13]. Proponents of full-scale analysis (also known as “shift”-analysis or “sliding dichotomization”) support its use by invoking Shannon’s seminal work on information systems and Altman’s and Royston’s work on the advantage of ordinal scale analysis vis-à-vis dichotomization [14]–[18]. Their central argument is that the loss of information inherent in switching from full-scale analysis to dichotomization may obscure important treatment effects [13], [19]. The ‘Shift’ approach as suggested by Saver and Gornbein [12] and used in SAINT I [20], SAINT II [21], and IST-3 [22] was conceived as the difference in distributions between treatment and control groups as an ordinal/categorical analysis of outcome classification across all ranks, grades or a major part of the ordinal scale. This ordinal scale analysis is similar to that suggested by Whitehead [7] and Campbell et al [8]. It assumes a common proportional odds ratio applied to mRS 0, mRS 0–1, mRS 0–2, mRS 0–3, etc. Note that this “shift’ differs from a change in modified Rankin score from baseline for each patient, as suggested by Lai and Duncan [23].

On both sides of this discussion (i.e., use of dichotomization vs. ‘shift’ analysis), there has not been explicit consideration of uncertainties regarding how well the recorded mRS scores reflect each patient’s true recovery state. However, from the work of van Sweiten et al and others we know that inter-rater reliability of mRS is relatively low [24]–[27], particularly for mid-range (mRS scores of 2–4) values. Quinn et al have also shown that uncertainties in mRS assessment persist in spite of certification and re-education of assessors and do not depend on the assessors’ field of specialization, educational background, country of origin, native language or length of patient interview [27]–[29]. These findings indicate that uncertainty or “noise” in the Rankin scoring may not be negligible, and indicate a need for closer examination of the patient-observer-score model that is the foundation of stroke outcome measurement.

In information processing terminology, dichotomization with an efficacy measure (mRS 0–1) and a safety measure (mRS 6) can be considered as an implementation of a band-stop filter. A central concept in information theory is the communication system which consists of a transmitter, a channel, and a receiver [15]. The transmitter produces a signal/symbol which is then passed on through the channel to the receiver for interpretation. In real-world situations, the channel is susceptible to noise which may corrupt the transmitted signal/symbol such that the receiver sees a different signal than was originally sent. This model is applicable to the situation of an observer evaluating a stroke patient, where the patient (transmitter) has a true Rankin score (signal) which is transmitted through the noisy channel of human assessment (observer) and is ultimately recorded as the outcome score for that patient (receiver).

In this paper, we hypothesized that uncertainties in assessment of this subjective outcome scale could be modeled and that errors will be higher if the entire scale is used compared to dichotomous measures. We calculate the error introduced by the channel (i.e., observer) during the transmission of the ordinal scale and dichotomized outcomes to an observer. Van Swieten’s inter-rater variability matrix in mRS classification by different observers is used as a characterization of the noise introduced by the observation channel [24]. The inter-rater variability matrix has been termed the ‘confusion matrix’ in various sub-fields of information theory [30]. Using the confusion matrix, the error rate for each approach was calculated. We then demonstrate the effect of the noise/error on sample size calculations using the SAINT I trial as our working example [20]. SAINT I is a particularly interesting test case because this earlier phase trial reported positive results with the “Shift” approach as the primary outcome measure, with unspecified positive dichotomizations. The SAINT trials tested a putative neuroprotectant, NXY-059, in acute ischemic stroke with hopes that it would improve outcome or reduce the hemorrhage rate after thrombolysis. While SAINT I was considered positive using a ‘shift’ analysis to compare the range of ordinal mRS scale 0 to 4 and collapsing scale 5 and 6 in treated patients vs. the placebo control group, the subsequent larger SAINT II trial did not demonstrate benefit with respect either to the “shift” or the commonly used mRS 0–2 dichotomous score [21]. We investigated whether increased error due to noise in the mRS indicated that the sample size targeted in SAINT I was smaller than calculated in the absence of noise. If true, then the likelihood for a spurious result is increased given an inadequate sample size.

## Methods

### Literature Search to Identify Stroke Randomized Clinical Trials

Two investigators (PM and JNS) independently performed structured searches in Medline to identify potentially eligible clinical stroke trials using keywords ‘acute’, ‘ischemic’, ‘stroke’ and ‘Rankin Scale (or Score)’ and reviewed all abstracts and retrieved articles for study inclusion. Studies were eligible for inclusion if they: 1) were randomized controlled stroke trials with at least 10 subjects in each study arm, 2) reported full range of mRS (0–6) outcome data in both the intervention and control group(s) at least 3 months or beyond, and 3) were published as original research manuscripts in English in a peer-reviewed journal. Two hundred and ninety-six articles were retrieved by this keyword search and subsequently reviewed, from which we identified 35 RCTs that met our inclusion criteria. Thirty-eight control arms from these 35 RCTs were then evaluated using our model to estimate misclassification error rates. For this study, we selected the control arms of these RCTs because sample size estimates for testing a novel treatment are calculated using the control arm ordinal scale outcome such as mRS along with treatment effect size [7].

### Misclassification Rates with Ordinal, Collapsed Ordinal and at Various Dichotomization and Trichotomization Cut-points

To calculate the misclassification or error rates in different scenarios a custom MATLAB® program was created. Error rates are computed in three sequential steps.

Step 1: For each of the 38 placebo/control arm distributions, simulated patient populations were generated and each patient’s mRS was stored as ‘mRS-Observed’. Due to wide variability in number of patients from 15 to >1500 in the trials, an arbitrarily large number (n = 10000) of patients were simulated as previously used in similar studies [31], [32]. A single one-step command ‘repmat’ is able to accomplish this task in Matlab®. This step essentially creates 10000 patients and reflects the mRS distribution reported for each trial. Each of these 10000 patients is then assigned a Rankin score, termed mRS-Observed(j) (see File S1)_._

**Figure 1:**
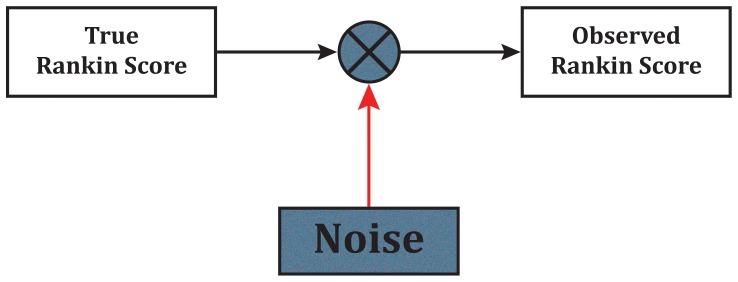
Shannon’s information transmission model adapted to scoring of a patient on the 7 point modified Rankin Scale. A noise or error source is assumed to be in the channel between the sender represented by the ‘True Rankin’ score and the receiver represented by the ‘Observed Rankin’ score.

Step 2. Results of this step were passed through the Shannon’s noisy channel model with van Swieten’s confusion matrix serving the role of noise (Figure 1). For example a patient may have been assigned a mRS grade of 2 in step 1 but due to the effect of noise in the system may be assigned an mRS grade other than 2. The output of this step for each patient is termed the “mRS-true”. At the end of this step, each of the 10000 patients will be assigned a “mRS-true(j)”.

Step 3. Misclassifications are counted for each patient when there is a mismatch between the input (mRS-Observed(j) by step 1) and output (mRS-true(j) after passing through Shannon’s noisy channel by step 2; Figure 1). The equation below summarizes this step for each subject ‘A(j)’.

A(j) is assigned a 1 if mRS_observed_(j) = mRS_true_(j) otherwise it is a 0.

Step 4. Total misclassification is then computed by summing across all subjects and dividing by the number of patients. This step is summarized in the form of an equation given below.
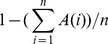



Misclassification/error percentages were calculated for all 38 control arms for different scenarios that have been used in various stroke trials: full range of mRS (i.e., a full shift analysis); collapsing the higher grades of mRS 4–6 into one grade and considering mRS 0,1,2,3 as independent grades; ‘dichotomizing’ at four different cut-point of mRS 1,2,3,4 and for two different trichotomizations (mRS 0–1, 2–4, 5–6 and mRS 0–2, 3–4, 5–6 [33]).

A user-driven MATLAB® program is provided in File S1 that takes the mRS 0–6 distribution of a control/standard treatment arm along with a user selected confusion matrix (default of van Swieten or user-entered) and provides error percentages for the full range of ordinal scale, collapsed scale, and various dichotomizations and trichotomizations. The equation is flexible and can accommodate scales with different number of categories.

Note that van Swieten’s inter-rater variability matrix was tabulated for the Rankin scale ranging from 0–5, while recent trials use the modified Rankin scale ranging from 0–6 with 6 representing death. Since there is likely low inter-rater variability in the diagnosis of death, a corresponding noise-free element was added to the van Swieten matrix.

### Sample Sizes for SAINT I Based on Consideration of mRS Errors

Sample sizes for full ordinal scale analysis is based on an assumption of a common proportional odds across the whole range [7], [8], [14], [32], [34]. Lees et al reported that they used a common proportional odds ratio (OR) of 1.3 to derive the sample size for SAINT I [20]. Whitehead [7] and Campbell et al [8] provided equations to calculate sample sizes when a common proportional odds ratio model is used with the full ordinal scale analysis [14]. Here, the common proportional odds model is applied to the placebo/control arm to derive the sample size [7], [8], [14].The equation provided by Campbell et al [8] and the initial equation of Whitehead [7] does not incorporate an error term for assessment of categories.

In the presence of an error in classification in ordinal scale analysis, Whitehead [7] provides an additional subsequent example to calculate sample sizes. This example requires that the complete distribution for the reference/control arm be available. Given that the distribution of subjects in the control arm of SAINT I for different grades of mRS is available, sample size was calculated using example worked out in Section 4 of Whitehead [7] but collapsing grades mRS 5 and 6 as was done in SAINT I [20]. Since the example provided by Whitehead is quite detailed, a custom MATLAB® implementation is provided in File S1.

### Other Statistical Tests

Tests of means were done by ANOVA routine supplied by Matlab®. Results of the ANOVA testing were used in post-hoc tests with a Matlab® routine ‘multcompare’. This routine implements Tukeys ‘honestly significant difference’ criterion [35].

## Results

### Error Rates

The placebo/control arms of the 35 trials were processed by steps described in the Methods section and error rates for different scenarios calculated. The median NIH stroke scale, a measure of baseline stroke severity from 0 (no deficit) to 38 (coma/dead), of the 35 trials with 38 control arms ranged from 3 to 24.

**Figure 2:**
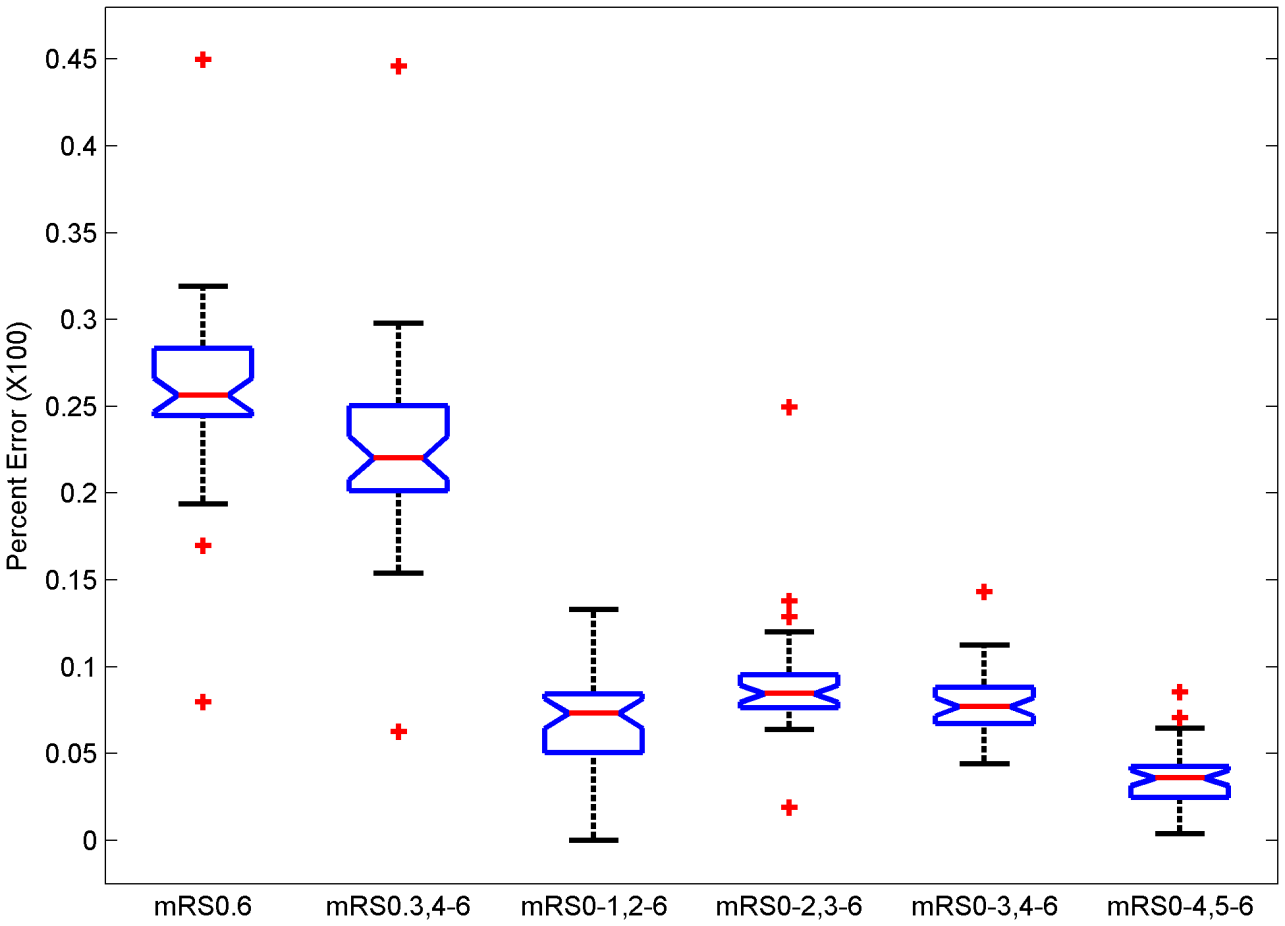
Box plots of error rates for the full ordinal scale of mRS (mRS 0.6), considering mRS 0 to 3 as individual grades and collapsing mRS grades 4 to 6 (mRS 0.3,4–6), dichotomizing at various cut-points of mRS 1 (mRS 0–1, 2–6), mRS 2 (mRS 0–2, 3–6), mRS 3 (mRS 0–3, 4–6) and mRS 4 (mRS 0–4, 5–6). van Swieten’s inter-rater reliability matrix used as confusion matrix. (p<.001 ANOVA; post-hoc testing shows that all dichotomization errors are lower than either full scale errors with mRS 0–4 dichotomization the lowest; p<.05).

If the full range of mRS is used, the misclassification error rates ranged from 7.8% to 44.4% (Table 1 and Figure 2; Mean±SD: 26.1%±5.31). Collapsing mRS grades 4 to 6 into one grade, as employed in the recently completed IST-3 trial [22] and considering the other grades as independent grades produced misclassification errors ranging from 5.9% to 44.0% (22.5%±5.66). If mRS 1 was chosen as the cut-off point, then the error rates ranged from 0% to 13.2% (6.8%±2.89). Error rates when using mRS 2 as a cut-off point were 1.7% to 24.8% (9.0%±3.33); for mRS 3 as a cut-off point the error rates ranged from 4.3% to 14.1% (7.8%±1.81); and for cut-off point of 4 the error rates ranged from 0.4% to 8.7% (3.5%±1.70). Comparison of means of error rates by ANOVA and post-hoc testing shows that the error rates were significantly different (p<0.0001) and all dichotomous errors lower than full range, with mRS 0–4 dichotomization error the lowest.

**Table 1:** Error percentages for 38 studies for the full ordinal scale (mRS 0.6), partially collapsed ordinal scale (mRS 0.3, 4–6) and dichotomization (mRS 0–1, 2–6; mRS 0–2, 3–6; mRS 0–3, 4–6; mRS 0–4, 5–6) and trichotomization (mRS 0–1, 2–4, 5–6; mRS 0–2, 3–4, 5–6) cut-points.

Study [Reference Number]	mRS 0.6	mRS0.3, 4–6	mRS0–1, 2–6	mRS0–2, 3–6	mRS0–3, 4–6	mRS0–4, 5–6	mRS0–1, 2–4, 5–6	mRS0–2, 3–4, 5–6
ABESTT [36]	25.64	23.37	9.41	8.62	6.24	2.27	11.68	10.89
ABESTTII [37]	23.19	20.8	8.04	7.6	6.18	2.39	10.43	9.99
ABESTTIICo [37]	27.26	24.8	9.29	9.77	6.99	2.46	11.75	12.23
ABESTTIIW [37]	31.48	29.73	9.39	14.64	7.68	1.75	11.14	16.39
ARTIS [38]	31.92	30.07	11.07	12.88	8.2	1.85	12.92	14.73
CAIST [39]	26.7	25.22	11.71	8.73	5.84	1.48	13.19	10.21
CASTA-Cereb [40]	30.4	27.44	9.39	11.49	8.24	2.96	12.35	14.45
Camerlingo [41]	24.71	18.09	5.54	7.26	6.2	6.62	12.16	13.88
Cereb-rt-pa [42]	23.45	21.94	9.73	7.3	6.07	1.51	11.24	8.81
DECIMAL [3]	7.76	5.93	0	1.68	4.25	1.83	1.83	3.51
DESTINY [4]	18.98	15.19	0	8.17	7.02	3.79	3.79	11.96
DP-b99 [43]	25.84	20.41	4.87	7.75	9.07	5.43	10.3	13.18
DP-b99-MACSI [44]	27.15	23.23	7.67	8.9	7.52	3.92	11.59	12.82
ECASSII [45]	25.4	22.53	6.48	8.59	8.2	2.87	9.35	11.46
ECASSIII [46]	22.09	19.37	8.93	6.56	4.96	2.72	11.65	9.28
EPITHET [47]	27.83	23.9	7.85	9.1	8.16	3.93	11.78	13.03
EPO [48]	26.13	20.76	7.08	7.1	7.42	5.37	12.45	12.47
Edaravone [49]	29.37	26.69	13.2	9.22	6.16	2.68	15.88	11.9
Enlimomab [50]	24.61	21.55	7.02	8.75	6.91	3.06	10.08	11.81
FIST [51]	24.14	20.39	5.15	9.33	7.03	3.75	8.9	13.08
GAIN [52]	26.19	21.55	5.61	8.02	8.93	4.64	10.25	12.66
HAMLET [5]	16.99	15.62	1.79	7.43	7.18	1.37	3.16	8.8
ICTUS [53]	25.14	21.24	4.85	8.53	8.93	3.9	8.75	12.43
IMS-III [54]	25.54	22.49	7.35	9.26	6.9	3.05	10.4	12.31
INSULINFARCT [55]	29.59	27.27	8.66	11.46	9.11	2.32	10.98	13.78
IST-3 [22]	23.05	18.38	6.31	7.56	5.52	4.67	10.98	12.23
MELT [56]	32.31	25.52	7.45	10.83	8.58	6.79	14.24	17.62
MR-RESCUE-Pen [57]	32.33	26.17	4.45	11.1	11.22	6.16	10.61	17.26
MR-RES-Non-Pen [57]	25.97	17.26	1.89	6.44	9.28	8.71	10.6	15.15
Minocycline [58]	44.37	43.99	6.99	24.82	14.12	0.38	7.37	25.2
NEST-1 [59]	27.22	22.53	7.2	8.53	7.43	4.69	11.89	13.22
NEST-2 [60]	29.17	25.13	5.69	10.94	9.84	4.04	9.73	14.98
NINDS [61]	23.96	20.57	6.19	7.88	7.45	3.39	9.58	11.27
PROACTII [62]	24.09	20.31	2.85	8.89	9.16	3.78	6.63	12.67
SAINTI [20]	25.08	21.71	7.6	7.13	7.96	3.37	10.97	10.5
SAINTII [21]	26.24	22.5	7.3	9.02	7.26	3.74	11.04	12.76
Synthesis [63]	25.16	22.32	5.02	7.32	10.25	2.84	7.86	10.16
Synthesis Exp [64]	26.51	22.88	7.12	8.18	8.35	3.63	10.75	11.81

**Figure 3:**
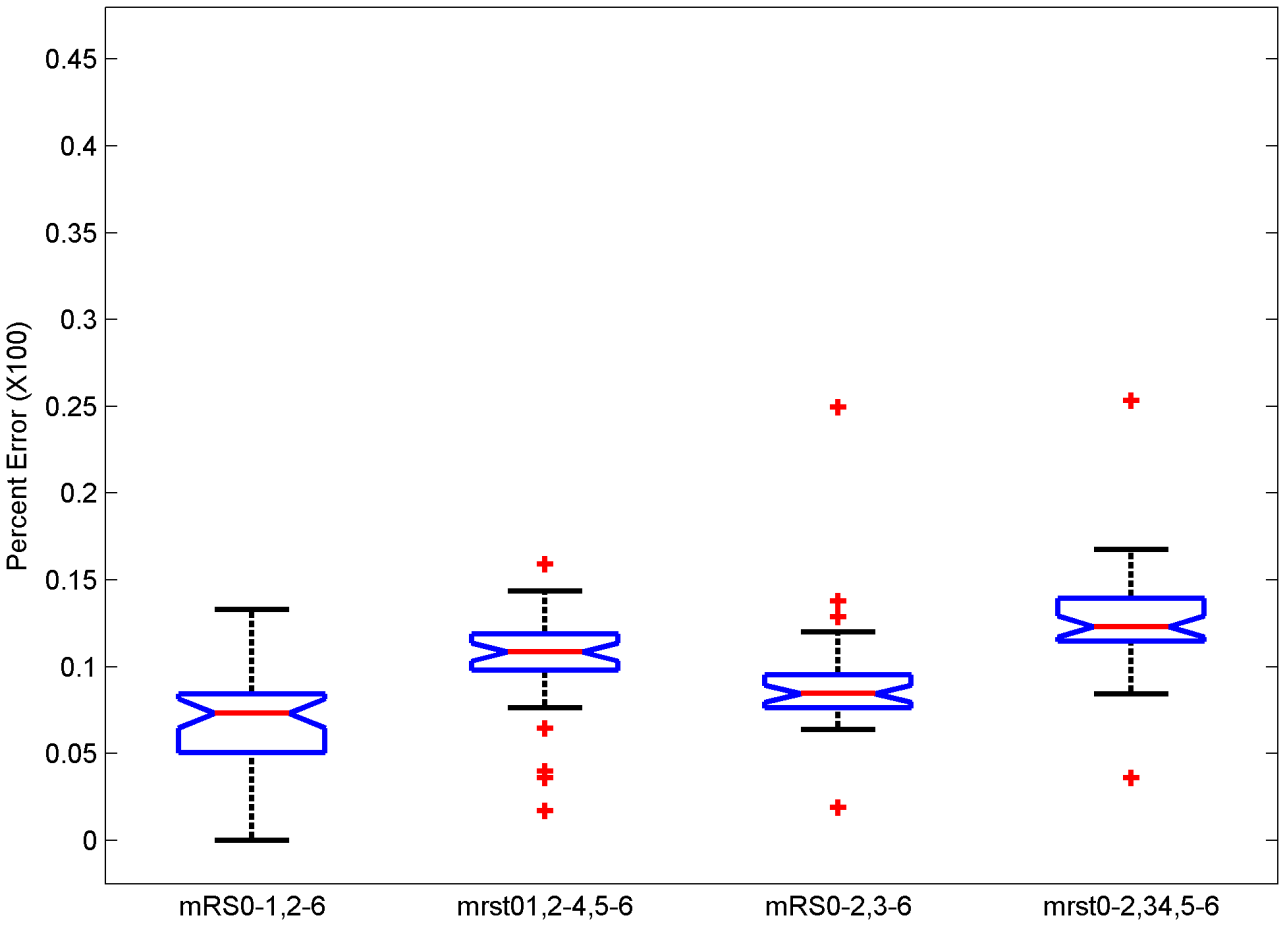
Box plots of error rates for dichotomizing at cut-point of mRS 1 (mRS 0–1, 2–6), trichotomizing at cut-points 1 and 4 (mRS 0–1, 2–4, 5–6), dichotomizing at mRS 2 (mRS 0–2, 3–6), and trichotomizing at cut-points 2 and 4 (mRS 0–2, 3–4, 5–6). van Swieten’s inter-rater reliability matrix used as confusion matrix. Post-hoc testing shows that both trichotomization errors are higher than dichotomization (p<.05).

Error rates for dichotomization mRS 0–1 and mRS 0–2 and two trichotomizations (mRS 0–1, 2–4, 5–6∶10.3%±2.75 and mRS 0–2, 3–4, 5–6∶12.6%±3.13) are shown in Figure 3. Post-hoc testing showed that the trichotomizations error rate was higher when compared to the corresponding dichotomization error (p<0.05).

There was a wide range of calculated error rates among the different trials, from 7.8%-44.4%. Error rate in DECIMAL trial [3] using the full scale mRS 0–6 was the lowest (7.8%). This is likely due to lower proportion of patients (22%) in the most uncertain grades (mRS 2–4) and the remaining (78%) being in a non-uncertainty- prone state of mRS 6 (i.e., deceased). Error rate in the Minocycline trial [58], for the full scale, was the highest (44.4%), possibly since only 14% were in the low uncertainty-prone grades (mRS 0–1), while, 81% were in the higher uncertain grades (mRS 2–4).

**Figure 4:**
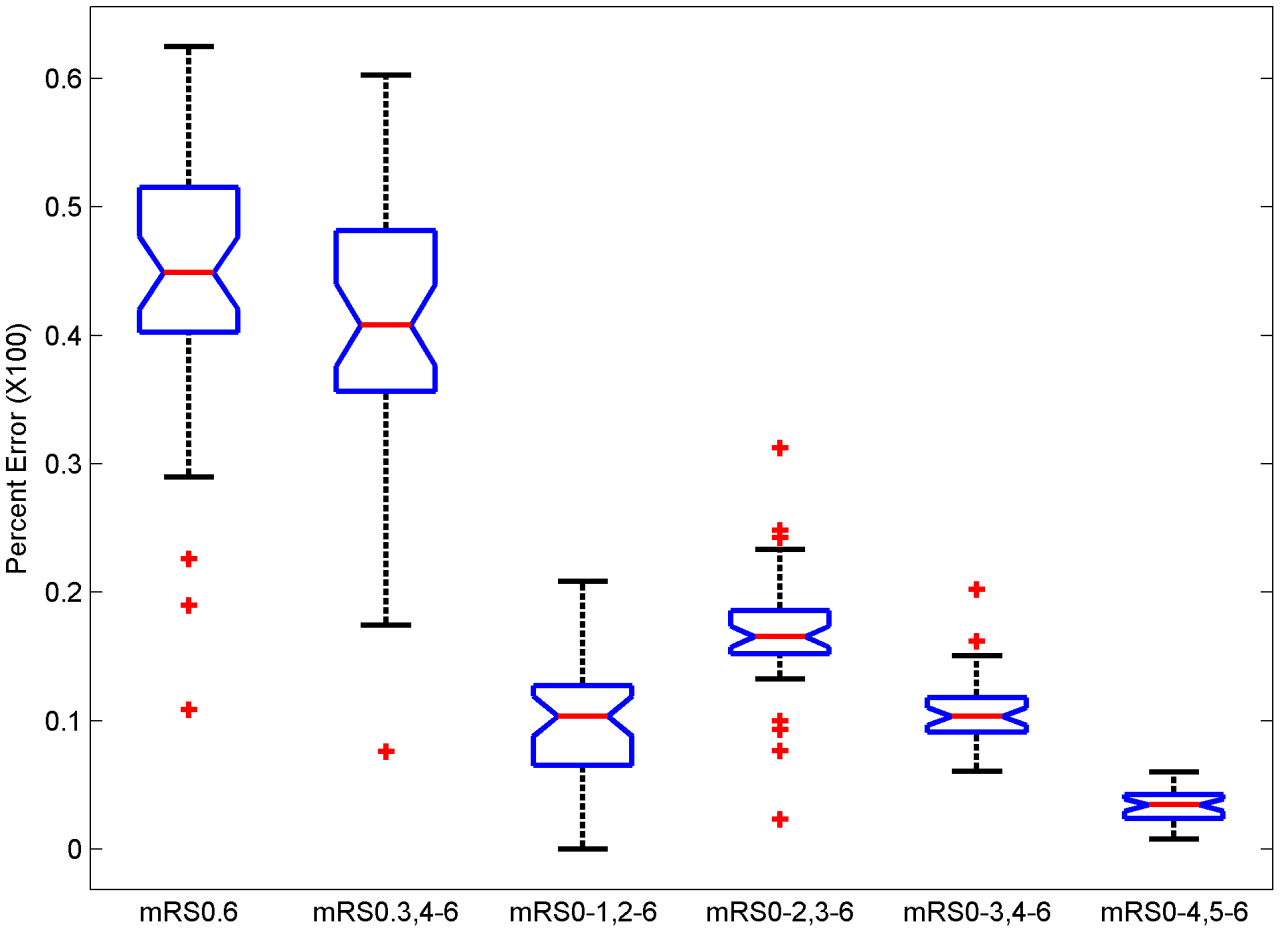
Inter-rater reliability matrix for mRS from Wilson et al [26] used as confusion matrix. Box plots of error rates for the full ordinal scale of mRS (mRS 0.6), considering mRS 0 to 3 as individual grades and collapsing mRS grades 4 to 6 (mRS 0.3,4–6), dichotomizing at a cut-point of mRS 1 (mRS 0–1, 2–6), dichotomizing at a cut-point of mRS 2 (mRS 0–2, 3–6), mRS 3 (mRS 0–3, 4–6) and mRS 4 (mRS 0–4, 5–6). Post-hoc testing shows that each error rate with this matrix is higher than the corresponding error using van Swieten’s confusion matrix except the error rates for mRS 4.

In place of van Swieten’s confusion matrix, the Wilson et al [26] matrix was applied and the above steps repeated resulting in higher error rates (Figure 4).

Post-hoc testing shows that each error rate with this matrix is higher than the corresponding error using van Swieten’s confusion matrix except that the error rates for mRS 4 dichotomizations have overlapping confidence intervals. Wilson et al proposed a modification of the mRS called the mRS-Structured Interview (mRS-SI) and also provided a confusion matrix [26]. The errors calculated with this confusion matrix were lower than with the original mRS, however, the errors for the full range and collapsed ranges are still significantly higher than the errors with dichotomization (p<0.001; data shown as Figure S2 in File S2).

### Calculation of Sample Size Incorporating mRS Measurement Errors

SAINT I [20] trial reported that, by applying ordinal analysis, the treatment arm showed efficacy vis-à-vis the placebo arm. A total of 1722 subjects were enrolled into two arms (861 in each arm) of SAINT I. However, applying the transformation matrix from van Swieten to account for misclassification and utilizing the available SAINT I placebo arm distribution,1070 subjects would be required in each arm to reliably estimate effects -nearly 24% more subjects than actually randomized (compare blue star to red star in Figure S3 in File S2). These calculations were repeated for SAINT II [21] employing their assumptions of a proportional odds ratio of 1.2 and power of 80%. SAINT II randomized 1621 patients to the placebo arm. If mRS misclassification was taken into account and using their mRS distribution, 1665 subjects would be needed, a difference of only 2.7%.

## Discussion

Clinical trials with subjective functional assessments have presented a variety of challenges. In the case of stroke, many clinical trial difficulties stem from issues such as heterogeneity of baseline factors, spontaneous recovery and subjective nature of assessing stroke severity and outcomes particularly given uncertainties in classification of outcomes [65], [66]. We show here that one such uncertainty, an asymmetrical distribution of misclassification in the mRS, introduces the need for more subjects to accommodate the potential biases in inferences about study effects that may occur if these uncertainties are not equally distributed**.** We include a set of Matlab programs (in File S1) that can be used in the future to estimate error rates and sample sizes using outcome scales. These programs are flexible in terms of categories and can be used with other outcome scales as long as the confusion matrix or equivalent is known. Note that while error estimates are important in estimating sample size, the lowest error configuration is not necessarily the best one if it does not capture the necessary range of expected outcomes. So for example, in a study of mild stroke, mRS 0–4, 5, 6 might be the lowest error, but miss important changes at the excellent outcome (mRS 0–1) range.

We performed an analysis of the influence of mRS misclassification on the expected error rates and applied this model to the empirical data derived from actual stroke clinical trials. We determined the influence of variability in mRS assessments on the overall misclassification error rates calculated for 38 individual control arms and showed that the error rates were highest when either the full-scale or collapsed full-scale (as in IST-3 [22], SAINT I [20], and SAINT II [21]) of mRS was considered as compared to dichotomization at cut-off points of mRS 1 and mRS 2. Using the SAINT I trial as an example, we demonstrated that when mRS misclassification uncertainties are taken into account, a higher sample size is required using the “shift” approach. Hence, SAINT I may have randomized 24% too few patients taking errors into consideration, thus, possibly accounting for the discrepant results between SAINT I and the larger SAINT II trial. There are other possible explanations for discrepant results between the two trials and we cannot prove that inadequate sample size was the primary factor. However, the larger SAINT II employed a lower proportional odds ratio of 1.2 with a lower power (80%) and there was a marginal difference of 2.7% between actual sample size and that required by taking into account misclassification in mRS.

The actual error rates found depend on the range of the mRS in each trial because the uncertainty in misclassification is not evenly distributed across the entire range. While there is considerable evidence that there is loss of information when a scale is dichotomized at the median [14], it is not clear that the advantage of use of the wider range will always overcome the noise that it appears to generate.

Our results echo the concept put forward by Whitehead [7], that the advantage of decreased sample size with ordinal scale is lost if there are errors even modest in classification. He calculated that a uniform error of 20% in a hypothetical four-category scale increased the sample size requirements by more than 60%. Whitehead’s projection was qualitatively confirmed here with real world mRS uncertainties and data derived from clinical trials. Misclassification of ordinal scale data leading to loss of power in statistical tests has been known for several decades [67].

It can be argued, from a strict information theory perspective, that misclassification error rates obtained by analyzing with the full-scale are not directly comparable with error rates obtained with the dichotomized approaches, since, there are different numbers of variables or “bits”. To address this potential criticism, a normalized error per bit of information transmitted (or entropy) was calculated [see details in File S2]. After normalization with entropy, rates, while overall lower, were still higher with full-scale analysis approach. Note, however, that entropy normalization reflects the error per bit of information transmitted, but does not influence the error factor that needs to be considered for sample size determination, that is the much higher value shown here.

The inter-rater reliability matrix proposed by van Swieten et al [24] was derived from an assessment of 100 patients by pairs of physicians selected from a pool of 34. These 34 physician raters were either senior neurologists or resident physicians. This situation may not reflect the actual clinical trial environment where typically there are 100 s of patient subjects and raters with various educational backgrounds spread across several continents [27]–[29]. Wilson et al [26] study used two neurologists, one stroke physician, seven nurses and four physiotherapists. The inter-rater reliability in the Wilson et al study was lower than van Swieten’s and resulted in higher error rates (Figure 4) when analyzed with the Shannon Entropy model compared to the van Swieten confusion matrix (Figure 2).

Other alternatives to van Swieten’s inter-rater reliability table are not without limitations. Some of these publications did not report evaluations at the lower and higher ends of mRS [25], [28], while others had fewer clinical assessors [25]–[27], [68], [69], and fewer patients [27], [68], [69] or, lacked face-to-face interviews. More attention needs to be given to the reliability of different implementation methods for rating outcomes, including centralized rating methods and incorporation of their errors into sample size estimation. Ideally, a comparison between a ‘typical’ assessor and a gold-standard ‘expert’ could be used. However, it is unclear if two ‘experts’ would agree on the assignment of a mRS grade to a patient given that studies on inter-rater reliability have reported kappa values ranging from 0.25 to 0.95 [27]. Additionally, evidence suggests that disagreement can still persist after training the typical assessor, and then, comparing his/her score against that of an expert [28]. Over the last decade there have been attempts at improving the reliability of mRS assessment with the aid of a structured interview [26], questionnaire [68], and a focused assessment [69], although replication of these improvements have been inconsistent [26], [29].

While our focus in this study has been the mRS, this same analysis can be extended to other ordinal scales employed in clinical trials. For example, Glasgow Outcome Scale (GOS), used in traumatic brain injury trials and infrequently in stroke trials, also demonstrate comparable error rates in the mid-range of the scale [70]–[72].

In conclusion, using stroke trials as an example, we demonstrated that misclassification error rates are overall higher with variations on the ‘shift’ analysis compared to dichotomization approach. We also demonstrated that the ‘shift’ analysis can lead to the need for higher sample size in the setting of misclassification. Selecting an appropriate sample size, while important, is difficult in the setting of uncertainties in measurement [73]. We found that in the case of mRS as the outcome measure, dichotomous outcomes are more reliable. Therefore, if ordinal analysis is employed, errors should be explicitly considered in sample size determination. In principle, we have outlined an approach to error estimation for any condition in which there are uncertainties in outcome assessment and provided the user with a set of Matlab programs to incorporate errors into sample size estimation. The relative advantage of dichotomizing vs. ordinal analysis will depend on the distribution of these uncertainties and the frequency of their occurrence under the specific conditions of the trial.

## Supporting Information

File S1 (DOCX)

File S2 (DOCX)
