# Supplementary material for: Quantification of Errors in Ordinal Outcome Scales Using Shannon Entropy: Effect on Sample Size Calculations
Source: PLoS One. 2013 Jul 5;8(7):e67754. doi: 10.1371/journal.pone.0067754 (PMC3702531; doi:10.1371/journal.pone.0067754)
Supplement: File S1 — (DOCX) [file pone.0067754.s001.docx]

Matlab program for Quantification of Error in categorical scales using Shannon Entropy

function maout = Shannon_be(ma, confusion_matrix, GOS_FLAG)

% Developed by Chase S. Krumpelman MD PhD MSEE

% Modified by Pitchaiah Mandava MD PhD MSEE

% This function developed for 7 grade mRS scale. However, it will work

% for a potential 6,7, and 8 grade scale.

%% Input

% User is expected to provide the name of the Study and the mRS

% distribution at the Matlab prompt '>'. Example provided below.

% > ma(1).Study = 'ABESTT';

% > ma(1).m = [13.5 26.5 13.0 16.0 13.5 5.0 12.5];

%

% If confusion_matrix (2nd argument) is not given by user then van Swieten

% matrix is loaded.

% Third argument is GOS_FLAG. Set it to a 1 if passing GOS/EGOS distribution

% and GOS or EGOS confusion matrix

% mRS grade 6 is deceased state but in GOS and EGOS grade 1 is deceased state

% If GOS_FLAG is set to a 1 then the distribution and confusion matrices

% are flipped left to right (a mirroring operation)

%% Output: The following numbers are printed out

% 1.Full range error; 2. Collapsed full range error (Grades 0..3, and collapsed

% top 3 grades); 3. Dichotomization error for Grades 0-1, 0-2, 0-3, 0-4

% if a 7 grade mRS scale is used. For the 8 grade EGOS an additional

% dichotomization error for grades 0-5 is printed.

% Two separate trichotomizations are also printed out Grades 0-1, 2-4, 5-6

% and 0-2, 3-4, and 5-6

% Output would look like this

% Study 0..6 0.3,4-6 0-1 0-2 0-3 0-4 Trich1 Trich2

% ABESTT 0.2564 0.2337 0.0941 0.0862 0.0624 0.0227 0.1168 0.1089

%% Load Van Swieten et al Confusion matrix by default if none loaded

if nargin ==1

confusion_matrix = [5 0 0 0 0 0 0; 0 6 2 0 0 0 0;...

1 4 13 5 2 0 0; 0 0 6 9 4 0 0; 0 0 0 2 8 1 0;...

0 0 0 0 8 24 0; 0 0 0 0 0 0 1]; GOS_FLAG = 0;

elseif nargin ==2

GOS_FLAG = 0;

elseif(nargin ==3) && (GOS_FLAG ==1)

confusion_matrix = fliplr(confusion_matrix); %For GOS flip around for vertical axis

confusion_matrix = flipud(confusion_matrix); % and a horizontal axis

end;

%%

for study_number = 1:1:size(ma,2)

study_name = ma(study_number).Study;

scale_used = length(ma(study_number).m)-1;

number_of_patients=10000;

%% Generate a roster of 10000 synthetic patients, and assign a Rankin Score

% generated from the study prior

if GOS_FLAG

study_prior = (fliplr(ma(study_number).m))./sum((fliplr(ma(study_number).m)));

else

study_prior = (ma(study_number).m)./sum(ma(study_number).m);

end;

% truth is the synthetic patients' ground truth Rankin score

truth_matrix = sw_sampler(repmat(study_prior,number_of_patients,1));

truth_labels = truth_matrix * [0:1:scale_used]';

% we know from van Swieten et al. that there is some distortion introduced

cm_norm = confusion_matrix./repmat(sum(confusion_matrix,2),1,size(confusion_matrix,1));

% we can perturb our ground truth according to van Swieten's observations

perturbed_truth_matrix = zeros(number_of_patients,scale_used+1);

experimental_confusion_matrix = zeros(scale_used+1);

for i=1:1:scale_used+1

indices = find(truth_matrix(:,i)==1);

% fprintf('found %d indices with label %d\n',length(indices),i);

perturbed_truth_matrix(indices,:)=sw_sampler(repmat(cm_norm(i,:),length(indices),1));

experimental_confusion_matrix(i,:)=sum(perturbed_truth_matrix(indices,:),1)./sum(sum(perturbed_truth_matrix(indices,:),1));

end

perturbed_truth_labels = perturbed_truth_matrix*[0:1:scale_used]';

% First, what is the baseline error?

number_misclassified = number_of_patients - sum(sum(truth_matrix.*perturbed_truth_matrix));

baseline_misclassification_rate = number_misclassified/number_of_patients;

%%

truth_matrix_0to3_Collapse_last_3(:,1:(scale_used-2)) = truth_matrix(:,1:(scale_used-2));

perturbed_truth_matrix_0to3_Collapse_last_3(:,1:(scale_used-2)) = perturbed_truth_matrix(:,1:(scale_used-2));

% Collapsing mRS 4, 5 and 6 together

truth_matrix_0to3_Collapse_last_3(:,(scale_used-1)) = truth_matrix(:,(scale_used-1))|...

truth_matrix(:,scale_used)|...

truth_matrix(:,(scale_used+1));

perturbed_truth_matrix_0to3_Collapse_last_3(:,(scale_used-1)) = perturbed_truth_matrix(:,(scale_used-1))|...

perturbed_truth_matrix(:,scale_used)|perturbed_truth_matrix(:,(scale_used+1));

collapsed_num_misclass = number_of_patients -...

sum(sum(truth_matrix_0to3_Collapse_last_3.*perturbed_truth_matrix_0to3_Collapse_last_3));

collapsed_misclass_rate = collapsed_num_misclass/number_of_patients;

%% calculate misclassification in bins for different cut-points

for cutpoint = 1:(scale_used-2)

bin_good_misclassification_count(cutpoint)=0;

bin_bad_misclassification_count(cutpoint)=0;

bin_dead_misclassification_count(cutpoint)=0;

for i=1:1:number_of_patients

if (perturbed_truth_labels(i) ~= truth_labels(i)) % if there is a misclassification

if ((perturbed_truth_labels(i)<=cutpoint)&(truth_labels(i)>cutpoint))

% this happens if we have put the patient in the "good outcome" bin, but actually they had a bad outcome

bin_good_misclassification_count(cutpoint) = bin_good_misclassification_count(cutpoint) + 1;

elseif (((perturbed_truth_labels(i)>cutpoint)&(perturbed_truth_labels(i)<scale_used))&...

((truth_labels(i)<=cutpoint)|(truth_labels(i)==scale_used)))

% this happens if we have put the patient in the "bad outcome" 2-5 bin, but actually they had a good outcome (or died)

bin_bad_misclassification_count(cutpoint) = bin_bad_misclassification_count(cutpoint) + 1;

elseif ((perturbed_truth_labels(i)==scale_used)&(truth_labels(i)~=scale_used))

bin_dead_misclassification_count(cutpoint) = bin_dead_misclassification_count(cutpoint) + 1;

% fprintf('death misclassfied ????\n');

end

end

end

ma(study_number).bin_good_outcome_misclassification(cutpoint) = bin_good_misclassification_count(cutpoint)/number_of_patients;

ma(study_number).bin_bad_outcome_misclassification(cutpoint) = bin_bad_misclassification_count(cutpoint)/number_of_patients;

ma(study_number).bin_death_misclassification(cutpoint) = bin_dead_misclassification_count(cutpoint)/number_of_patients;

end;

% misclassification rate is how often an observer gave a label other than the "true" label

% fprintf(' For this dataset, the misclassification rate is %f\n',baseline_misclassification_rate);

ma(study_number).misclassification_rate = baseline_misclassification_rate;

ma(study_number).collapsed_misclass_rate = collapsed_misclass_rate;

% Trichotomization cutpoints (These are fixed)

for trik = 1:2

if trik == 1

c1 = scale_used-5; c2=scale_used-2;

else

c1 = scale_used-4; c2=scale_used-2;

end;

bin_good_but_actually_bad_misclas_count(trik)=0;

bin_good_but_actually_worse_misclas_count(trik) = 0;

bin_bad_but_actually_good_misclas_count(trik)=0;

bin_bad_but_actually_worse_misclas_count(trik) = 0;

bin_worse_but_actually_bad_misclas_count(trik) = 0;

bin_worse_but_actually_good_misclas_count(trik) = 0;

for i=1:1:number_of_patients

if (perturbed_truth_labels(i) ~= truth_labels(i)) % if there is a misclassification

if ((perturbed_truth_labels(i)<=c1)&...

(truth_labels(i)>c1)&...

(truth_labels(i)<=c2))

% this happens if we have put patient in "good outcome" bin, but actually patient a bad outcome

bin_good_but_actually_bad_misclas_count(trik) = bin_good_but_actually_bad_misclas_count(trik) + 1;

elseif ((perturbed_truth_labels(i)<=c1)&...

(truth_labels(i)>c1)&...

(truth_labels(i)>c2))

% this happens if we have put the patient in the "good

% outcome", but actually they had a worse outcome

bin_good_but_actually_worse_misclas_count(trik) = bin_good_but_actually_worse_misclas_count(trik) + 1;

elseif ((perturbed_truth_labels(i)>c1)&...

(perturbed_truth_labels(i)<=c2)&...

(truth_labels(i)<=c1)&...

(truth_labels(i)<=c2))

bin_bad_but_actually_good_misclas_count(trik) = bin_bad_but_actually_good_misclas_count(trik) + 1;

% this happens if we have put the patient in the "bad

% outcome", but actually they had a good outcome

elseif ((perturbed_truth_labels(i)>c1)&...

(perturbed_truth_labels(i)<=c2)&...

(truth_labels(i)>c2))

bin_bad_but_actually_worse_misclas_count(trik) = bin_bad_but_actually_worse_misclas_count(trik) + 1;

% this happens if we have put the patient in the "bad

% outcome", but actually they had a worse outcome

elseif ((perturbed_truth_labels(i)>c2)&...

(truth_labels(i)>c1)&...

(truth_labels(i)<=c2))

bin_worse_but_actually_bad_misclas_count(trik) = bin_worse_but_actually_bad_misclas_count(trik) + 1;

% this happens if we have put the patient in the "worse

% outcome", but actually they had a bad outcome

elseif ((perturbed_truth_labels(i)>c2)&...

(truth_labels(i)<=c1)&...

(truth_labels(i)<c2))

bin_worse_but_actually_good_misclas_count(trik) = bin_worse_but_actually_good_misclas_count(trik) + 1;

% this happens if we have put the patient in the "worse

% outcome", but actually they had a good outcome

end

end

end

ma(study_number).total_misclassification(trik) =...

(bin_good_but_actually_bad_misclas_count(trik)+...

bin_good_but_actually_worse_misclas_count(trik)+...

bin_bad_but_actually_good_misclas_count(trik)+...

bin_bad_but_actually_worse_misclas_count(trik)+...

bin_worse_but_actually_bad_misclas_count(trik)+...

bin_worse_but_actually_good_misclas_count(trik))/number_of_patients;

end;

end % end of loop over study_numbers

%%

String1 = ' Study 0..6 0.3,4-6 0-1 0-2 0-3 0-4 Trich1 Trich2\n';

fprintf(String1);

for i = 1:length(ma)

maout(i).Study=ma(i).Study;

maout(i).fullscale_error = ma(i).misclassification_rate;

maout(i).collapsed_full_error= ma(i).collapsed_misclass_rate;

fprintf('%20s %-10f %-10f', maout(i).Study,maout(i).fullscale_error,...

maout(i).collapsed_full_error);

for ii = 1: (scale_used-2)

maout(i).Dich_error(ii) = ma(i).bin_good_outcome_misclassification(ii)...

+ ma(i).bin_bad_outcome_misclassification(ii);

fprintf(' %-10f', maout(i).Dich_error(ii));

end;

maout(i).Trich1_error = ma(i).total_misclassification(1);

maout(i).Trich2_error = ma(i).total_misclassification(2);

fprintf(' %-10f %-10f\n', maout(i).Trich1_error, maout(i).Trich2_error);

end;

%%

function y=sw_sampler(x);

% function y=sw_sampler(x);

% where:

% x is a vector of bin probabilties (sum(x)==1)

% y is a random assignment according to the bin probabilities

location = repmat(rand(size(x,1),1),1,size(x,2))>cumsum(x,2);

y=zeros(size(x));

for i=1:1:size(x,1)

y(i,min(find(location(i,:)==0)))=1;

end

Matlab program for Sample Size calculation incorporating misclassification

function n_mis = d_calc_mis_whitehead(p_iC, theta_R, power, smis_class, amis_class, fid)

% Whitehead J Example 2 in Sample size calculations for ordered

% categorical data. Statistics in Medicine 1993:12:2257-2271

% Matlab implementation done by Pitchaiah Mandava MD PhD MSEE

% p_iC is an array of probabilities (or proportions) in control arm

% categories

% theta_R is the odds ratio

% Whitehead had common misclassification rate in all categories

% In our case the mis-classification rate is different for each

% category and is made up of two parts amis_class and smis_class.

% amis_class is the misclassification that is added to each category

% and comes from other categories. For example amis_class for mRS 2

% would be the percentage that should have been mRS2 but was

% mistakenly identified as mRS 0,1,3,4,5.

% smis_class is the percentage is subtracted from each category

% because a patient was mis-classified into a particular mRS 2

% while the patient should have been in a different category.

%

% smis_class is the subtraction from each category due to error

% amis_class is the addition into each category due to error

% Both are derived from van Swieten confusion matrix

% fid is file id where the program output is printed (1 is terminal)

%

% EXAMPLE

% d_calc_mis_whitehead(SAINT_I_6, log(1.3), .90, smis_class, amis_class,1)

% where SAINT_I_6 = [0.11 0.20 0.117 0.127 0.206 0.24]; % Reported mRS

% proportions in control arm of SAINT I

%

% log(1.3) is the log(Odds Ratio) % Reported log odds ratio in SAINT I

% .90 corresponds to 90% power

% smis_class and amis_class come from van Swieten Matrix

% smis_class = [0.083 0.325 0.431 0.483 0.457 0.0447];

% amis_class = [0 0 0.020 0 0 0

% 0 0 0.127 0 0 0

% 0.083 0.325 0 0.315 0.045 0

% 0 0 0.243 0 0.182 0

% 0 0 0.040 0.168 0 0.0447

% 0 0 0 0 0.227 0];

% Note that the column sums of amis_class equal smis_class

%%

Q_iC = cumsum(p_iC);

if Q_iC(length(Q_iC))>1

error('Cummulative proportion >1'); % Error Check

end;

C_len = length(p_iC);

%%

one_power = 1 - power;

%%

s_p_iC = p_iC.*smis_class;

a_p_ic = p_iC*(amis_class)';

p_iC_u0 = p_iC -s_p_iC+a_p_ic;

%%

Q_iC_u0 = cumsum(p_iC_u0);

if Q_iC_u0(length(Q_iC_u0))>1

error('Cummulative proportion >1');

end;

% Log odds ratio from formula for theta_R

Q_iER = Q_iC./(Q_iC + (1-Q_iC)*exp(-theta_R));

if Q_iER(length(Q_iER))>1

error('Cummulative proportion >1');

end;

p_iER = R_cumsum(Q_iER);

%%

R_len = length(p_iER);

s_p_iER = p_iER.*smis_class;

a_p_iER = p_iER*(amis_class)';

p_iER_u0 = p_iER -s_p_iER+a_p_iER;

%%

Q_iER_u0 = cumsum(p_iER_u0);

if Q_iER_u0(length(Q_iER_u0))>1

error('Cummulative proportion >1');

end;

%% Calculate theta_R_u0. Empty the last OR since it produces infinity

num_theta_R_u0 = (Q_iER_u0./(1-Q_iER_u0));

num_theta_R_u0(R_len) = [];

denom_theta_R_u0 = (Q_iC_u0./(1-Q_iC_u0));

denom_theta_R_u0(C_len) = [];

theta_R_u0 = log(num_theta_R_u0./denom_theta_R_u0);

%% Calculation of weights proportional odds are no longer satisfied.

ave_Q_i_u0 = (Q_iC_u0 + Q_iER_u0)/2;

wt_ave_Q_i_u0 = ave_Q_i_u0.*(1-ave_Q_i_u0);

wt_ave_Q_i_u0(R_len) = []; % empty the last weight since it is a zero

weighted_theta_R_u0 = sum(wt_ave_Q_i_u0.*theta_R_u0)/sum(wt_ave_Q_i_u0);

%%

% average probabilities

ave_p_i_u0 = (p_iC_u0+p_iER_u0)/2;

% 1-ave_p_i^3

one_ave_p_i_u0_3 = 1-sum(ave_p_i_u0.^3);

z_1_alpha_d_half = max(norminv([0.025 (1-0.025)]));

% Power

z_beta = max(norminv([one_power (1-one_power)]));

%%

n_mis = (3*4*(z_1_alpha_d_half+z_beta)^2)...

/(((weighted_theta_R_u0)^2)*one_ave_p_i_u0_3);

fprintf(fid, 'Sample size with misclassification %d\n', round(n_mis));

function [ output_arg ] = R_cumsum( input_arg )

% Get categorical values from cumulative probabilities/

% may work with vectors only.

vl = length(input_arg);

output_arg(1) = input_arg(1);

for i = 2: vl

output_arg(i) = input_arg(i)-input_arg(i-1);

end
