# Supplementary material for: Quantification of Errors in Ordinal Outcome Scales Using Shannon Entropy: Effect on Sample Size Calculations
Source: PLoS One. 2013 Jul 5;8(7):e67754. doi: 10.1371/journal.pone.0067754 (PMC3702531; doi:10.1371/journal.pone.0067754)
Supplement: File S2 — (DOCX) [file pone.0067754.s002.docx]

**Entropy in full range and dichotomization of mRS for efficacy and safety**

Entropy of an information system is given by the equation (Cover and Thomas [16] equation 1.1)

H= - [p0*log_2_(p0) + p1*log_2_(p1) + p2*log_2_(p2) + … + pn*log_2_(pn)] ---- 1

where pn is the proportion of subjects or events in a particular state or grade n. With the assumption that all states or grades are equally likely, equation 1 can be simplified to

H = -log_2_(number of states) ---------------------------------2

The full Rankin scale contains 7 unique states, and our dichotomized model contains 3 states. Efficacy measures in most trials are reported as proportion of subjects that achieve a functional state either below (state 1: mRS 0-1) or above a cut-off point (state 2: mRS 2-5). Additionally, safety measures are reported as mortality data (state 3: mRS 6). The entropy value obtained by equations 1 and 2 is also the amount of information in a system, or using Tukeys nomenclature: bits in a system.

With the assumption that every state is equally likely, the entropy calculation for the seven states of the complete mRS 0-6 is

H_7 = log_2_(7) = 2.8074

Recently the IST3 trial considered mRS 0,1,2 and 3 as separate states and grouped mRS 4, 5, 6 together thus creating five states. The entropy calculation for five states is

H_5 = log_2_(5) = 2.3219

And similarly, for the three states of efficacy dichotomization and mortality, it is

H_3 = log_2_(3) = 1.5850

We were interested in comparing the error rates incurred by transmitting the full mRS range vs. the dichotomized models. These models have different numbers of states and hence their misclassification rates are not directly comparable and must be normalized. The misclassification (error) per bit of entropy in the source was normalized. Error information for full range and dichotomization was recalculated as a measure of error per bit of information.

Normalizing the error rates in terms of entropy or information per bit, the full range of mRS 0-6 has an error rate of 9.0%±1.66, the five state mRS 0..3,4-6 has error of 9.5%±1.95 and dichotomization at cut-point of mRS 1 has an error rate of 4.2%±1.93, at cut-point of mRS 2 has an error rate of 5.5%±1.42, at cut-point mRS 3 error rate is 4.74%±1.00 and at cut-point of mRS 4 error rate is 2.2%±0.99 (p <0.0001 by ANOVA; Figure S1). The error per bit of information for the five state as used in IST3 is higher (Figure S1) since the entropy (or the information content) is lower for five states.


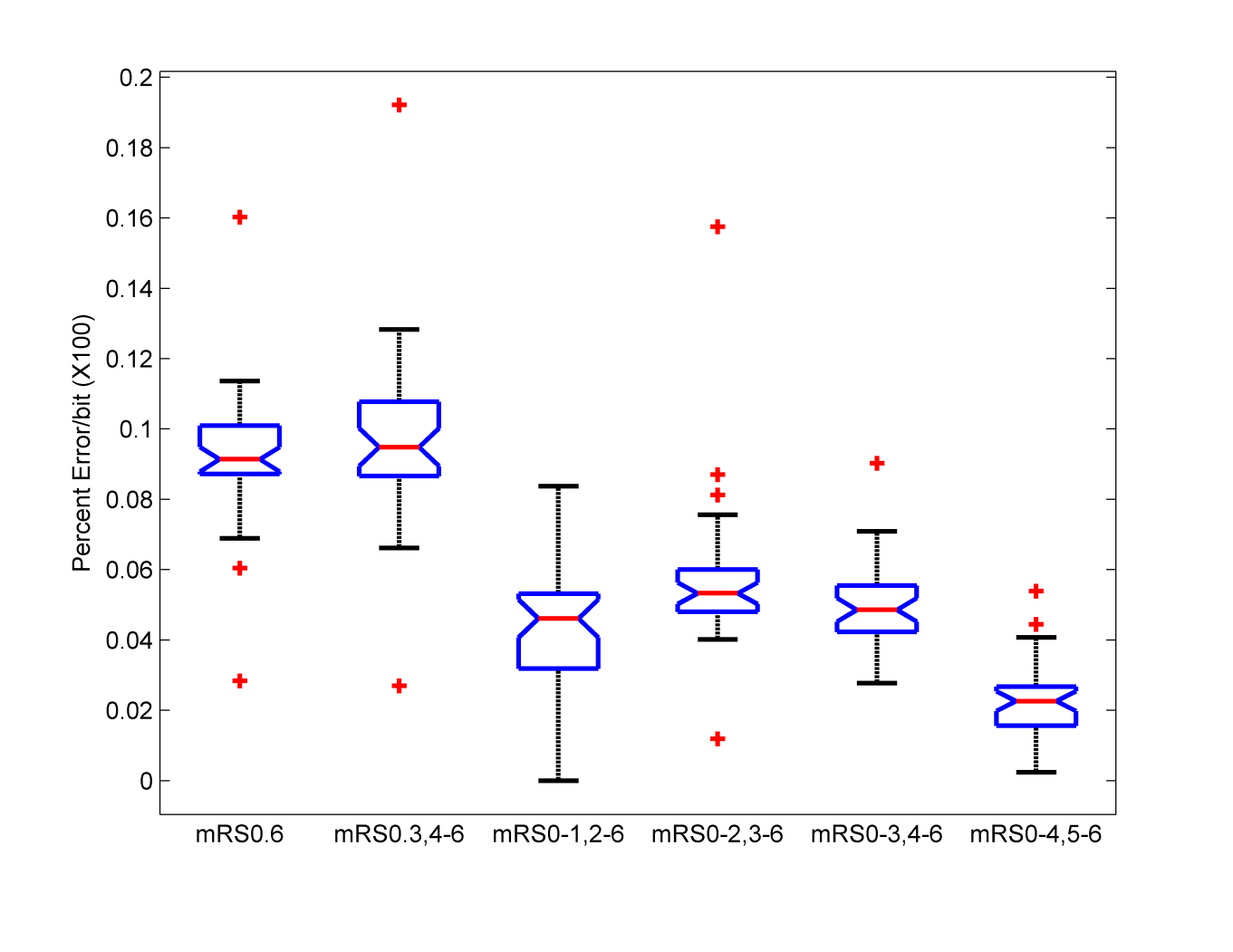
**Figure S1**. Box-plots of percent error rate per bit of information for the full range of mRS, collapsed mRS and with **four** different cut-points for dichotomization (p<0.001 by ANOVA).


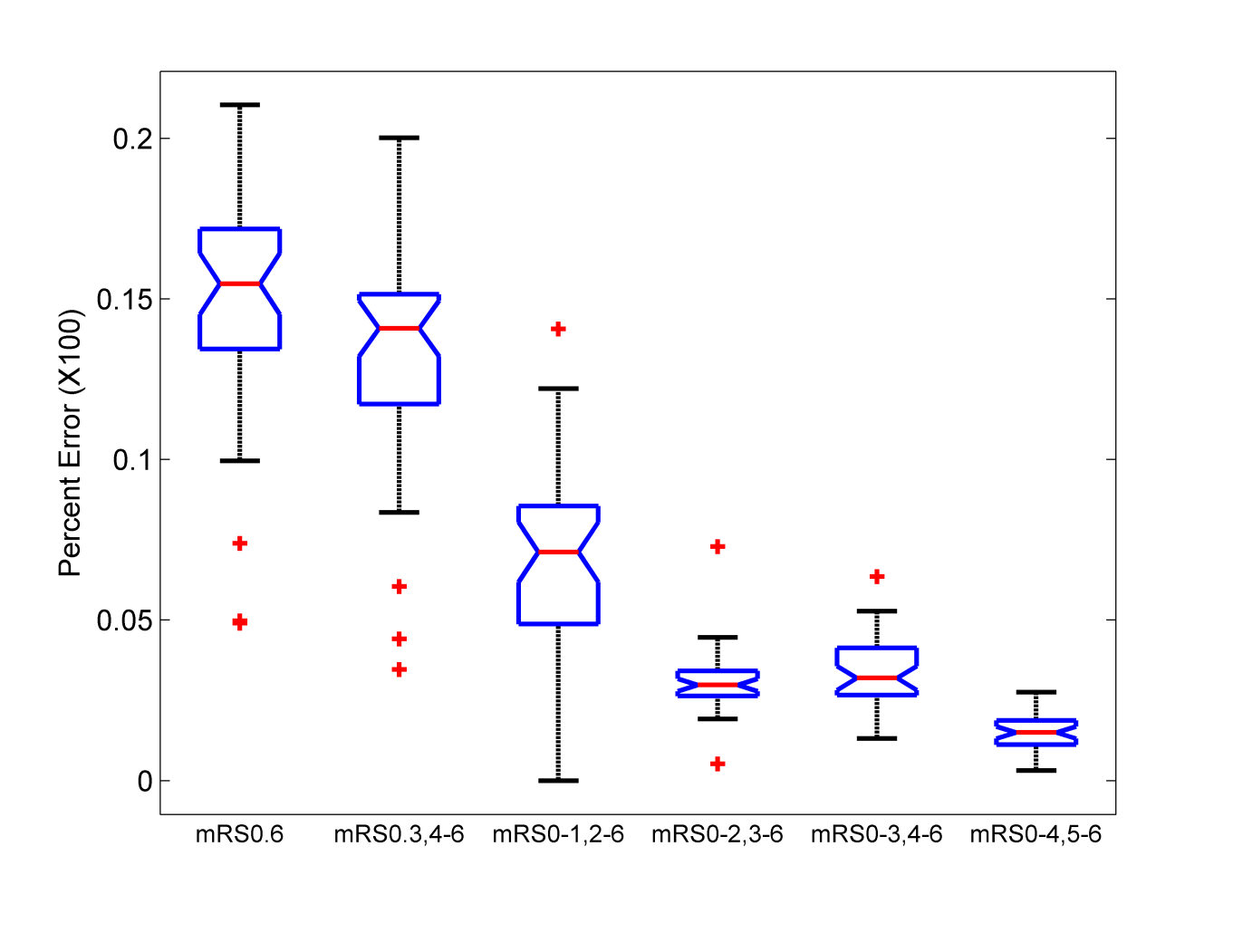


**Figure S2.** mRS-Structured Interview (mRS-SI) Confusion matrix from Wilson et al used to calculated error in six scenarios. Box-plots of percent error for the full range of mRS, collapsed mRS and with four different cut-points for dichotomization (p<0.001 by ANOVA).


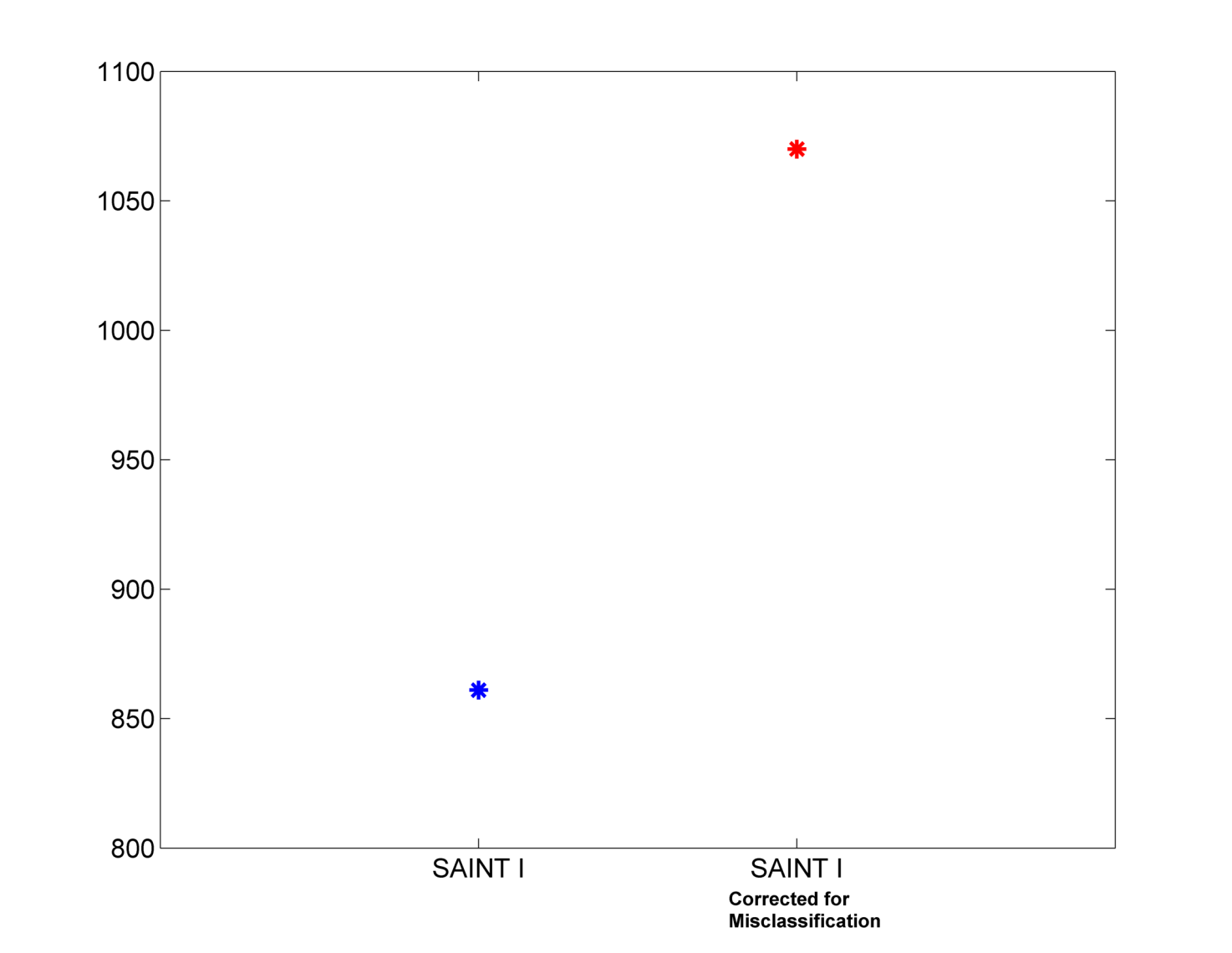


**Figure S3**. SAINT-I published and recalculated sample sizes taking mRS misclassification errors into account.
